# Prospective Study on Prophylactic Micafungin Sodium against Invasive Fungal Disease during Neutropenia in Pediatric & Adolescent Patients Undergoing Autologous Hematopoietic Stem Cell Transplantation

**DOI:** 10.3390/children9030372

**Published:** 2022-03-07

**Authors:** Bo-Kyung Kim, Jung-Yoon Choi, Kyung-Taek Hong, Hong-Yul An, Hee-Young Shin, Hyoung-Jin Kang

**Affiliations:** 1 Department of Pediatrics, College of Medicine, Seoul National University, Seoul 03080, Korea; imkbk@snuh.org (B.-K.K.); choijy@snu.ac.kr (J.-Y.C.); hongkt@snu.ac.kr (K.-T.H.); anongg@snu.ac.kr (H.-Y.A.); hyshin@snu.ac.kr (H.-Y.S.); 2 Cancer Research Institute, Seoul National University, Seoul 03080, Korea; 3 Wide River Institute of Immunology, Hongcheon 25159, Korea

**Keywords:** micafungin, invasive fungal disease, autologous hematopoietic stem cell transplantation, pediatric, adolescent

## Abstract

Background: Invasive fungal diseases (IFDs) increase the mortality rate of patients with neutropenia who receive chemotherapy or have previously undergone hematopoietic stem cell transplantation (HSCT). Micafungin is a broad-spectrum echinocandin with minimal toxicity and low drug interactions. We therefore investigated the efficacy and safety of prophylactic micafungin in pediatric and adolescent patients who underwent autologous HSCT. Methods: This was a phase II, prospective, single-center, open-label, and single-arm study. From November 2011 to February 2017, 125 patients were screened from Seoul National University Children’s Hospital, Korea, and 112 were enrolled. Micafungin was administered intravenously at a dose of 1 mg/kg/day (maximum 50 mg/day) from day 8 of autologous HSCT until neutrophil engraftment. Treatment success was defined as the absence of proven, probable, or possible IFD up to 4 weeks after therapy. Results: The study protocol was achieved without premature interruption in 110 patients (98.2%). The reasons interrupting micafungin treatment included early death (*n* = 1) and patient refusal (*n* = 1). Treatment success was achieved in 109 patients (99.1%). Only one patient was diagnosed with probable IFD. No patients were diagnosed with possible or proven IFD. In the full analysis set, 21 patients (18.8%) experienced 22 adverse events (AEs); however, all AEs were classified as “unlikely” related to micafungin. No patient experienced grade IV AEs nor discontinued treatment, and none of the deaths were related to micafungin. Conclusions: Our study demonstrated that micafungin is a safe and effective option for antifungal prophylaxis in pediatric patients who underwent autologous HSCT, with promising efficacy without significant AEs.

## 1. Introduction

Invasive fungal disease (IFD) is an important cause of illness and death in patients with neutropenia, which is a condition that can often occur as a result of chemotherapy or hematopoietic stem cell transplantation (HSCT) [1,2]. The risk of infection is associated with the degree and duration of neutropenia, the disruption of protective skin and mucosal surface barriers, and the use of corticosteroids. A previous study reported a case fatality rate of 86.7% for allogeneic or autologous HSCT recipients with invasive aspergillosis [3]. Pediatric patients who underwent allogeneic or autologous HSCT showed an IFD incidence rate of 3–21% [4,5]. Patients who have previously undergone autologous HSCT are known to have a lower risk of IFD than allogeneic HSCT recipients. However, certain autologous recipients at a high risk benefit from prophylaxis of IFD [6]. Furthermore, several pediatric patients who underwent autologous HSCT died owing to IFD [7]. To reduce the high mortality rate associated with IFD, early initiation of antifungal therapy in patients with HSCT is required. However, studies on the development of effective antifungal agents with reduced interactions with HSC-related drugs have not been well established. A high crude mortality rate associated with IFD stems in part from difficulties in making a timely diagnosis. High-risk patients typically have a decreased inflammatory response and clinical features, which may not manifest before the infection is far advanced [8]. For many years, conventional microbiological, histological, and radiological techniques have been used for diagnosis; however, these techniques have a limited impact on clinical decision making, as they are time consuming and lack sensitivity and general accessibility. With the development of an enzyme immunoassay directed against *Aspergillus galactomannan*, the serological kits available for the detection of fungal antigens have shown inconsistent sensitivity, specificity, or both [9].

In view of these findings, an empirical strategy for the use of antifungal agents has been advocated since the early 1980s. Meanwhile, a presumptive strategy was chosen to treat invasive fungal infections during the neutropenic period. Hence, anti-*Aspergillus* agents were provided against persistent febrile neutropenia based on positive serum test and/or infiltrates or nodules on radiography or computed tomography [10]. In high-risk patients, such as those with leukemia or HSCT recipients, antifungal agents are initiated at a period of high risk of infection to prevent fungal infections.

Echinocandins are a novel class of antifungal agents that demonstrate antifungal activity against *Candida* and *Aspergillus* species [11]. Micafungin, a member of echinocandin, is a broad-spectrum antifungal agent and is associated with minimal toxicity and low potential for drug interactions [12]. It has mostly been used for the treatment of invasive candidiasis in pediatric patients. As previous studies have not reported any AEs associated with the use of micafungin, it is well tolerated in pediatric patients, including neonates [13,14].

There have been a few reports describing the prophylactic use of micafungin in pediatric patients. An early comparative, double-blinded, randomized phase III trial showed promising efficacy and safety of micafungin for prophylaxis in 386 adults and 39 pediatric patients who underwent HSCT [15]. However, the sample size of the pediatric group in this study was small, and the enrolled population was heterogeneous, including patients who had previously undergone autologous and allogeneic HSCT. A recent Japanese study suggested that prophylactic micafungin might prevent IFD in pediatric patients receiving allogeneic or autologous HSCT [16]. This study was a retrospective, single-center study, which enrolled only 14 patients. Park et al. [17] demonstrated the efficacy and safety of micafungin for the prevention of IFD in allogeneic HSCT in pediatric and adolescent patients.

Therefore, we aimed to investigate the efficacy and safety of micafungin as a prophylactic antifungal therapy, specifically in pediatric and adolescent patients who had previously undergone autologous HSCT.

## 2. Methods

### 2.1. Patients

Pediatric or adolescent patients (aged <21 years) with hematological and non-hematological diseases who had previously undergone autologous HSCT, including second autologous HSCT, were eligible for this study. The exclusion criteria were as follows: (1) aspartate transaminase or alanine transaminase level >5 times the upper limit of normal; (2) bilirubin >2.5 times the upper limit of normal; (3) a history of allergy, sensitivity, or any serious reaction to an echinocandin; (4) IFD at the time of enrollment; and (5) systemic antifungal therapy within 72 h before administration of the first dose of the study drug.

### 2.2. Study Design

**Figure 1:**
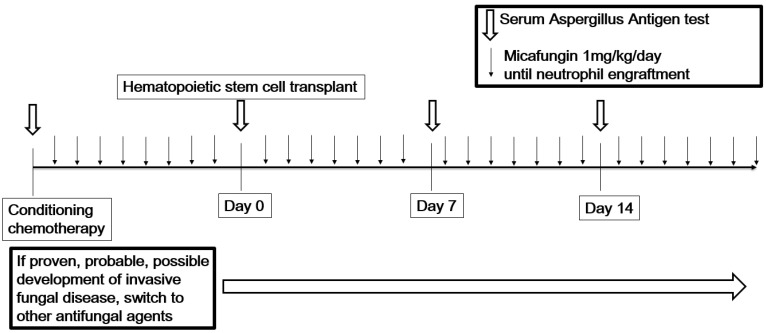
Treatment scheme of the study.

This was a phase II, prospective, single-center, open-label, and single-arm study. This study was approved by the Institutional Review Board of Seoul National University Hospital (No. 1102-038-351), and informed consent was obtained from the parents. This study was registered at clinicaltrials.gov (#NCT01417169). Eligible patients who provided informed consent were administered a single 1 h infusion of micafungin (Astellas Pharma US Inc., Deerfield, IL, USA) at 50 mg/day (1 mg/kg/day for patients weighing <50 kg). Infusion of micafungin was initiated on day 8 of autologous stem cell transplantation (Figure 1).

Patients received micafungin until the earliest of the following: (1) absolute neutrophil count (ANC) >1000/µL after the nadir absolute count; (2) development of proven, probable, or possible invasive fungal infections; (3) development of unacceptable drug toxicity; (4) death; (5) withdrawal from study participation (patient’s decision); or (6) discontinuation of study treatment (investigator’s decision). Serum galactomannan was sampled weekly (±3 days) from the day of initiation of micafungin administration. Patients terminating micafungin treatment owing to the above criteria (3)–(5) were considered as premature interruption.

### 2.3. Outcome

The intention-to-treat (ITT) set was defined as all patients who received at least 1 dose of micafungin. The primary endpoint was treatment success, which was defined as the absence of proven, probable, or possible IFD during the period of prophylactic therapy and up to 4 weeks after the end of micafungin administration in patients who completed the treatment according to the study protocol, including patients who switched to other antifungal agents by investigator’s decision. The primary endpoint efficacy set was defined as the number of patients who completed the treatment according to the protocol without premature interruption. The secondary endpoints were IFD-related mortality and safety profiles in the ITT full-analysis group.

Engraftment was defined as achieving an ANC > 0.5 × 10^9^/L for three consecutive days before day 28. Platelet recovery was defined as achieving a platelet count >20,000/mL, unsupported by platelet transfusions for 7 days.

Adverse events (AEs) were monitored throughout the course of therapy. Laboratory evaluations were conducted once a week during micafungin therapy. All AEs, except for generally expected AEs after autologous HSCT, were graded according to the Common Terminology Criteria for Adverse Events version 4.0 and rated as non-assessable, conditional, unlikely, possible, probable, and certain according to the World Health Organization—Uppsala Monitoring Center causality assessment system [18].

### 2.4. Definition of IFD

Proven, probable, or possible IFD is defined as described by the European Organization for Research and Treatment of Cancer/Invasive Fungal Infections Cooperative Group and the National Institute of Allergy and Infectious Diseases Mycoses Study group criteria (2008) [19]. Proven IFD is defined as detection of fungal elements in biopsy specimens or cultures of supposedly sterile materials or blood. Probable IFD is defined as detection of fungal elements directly or indirectly (galactomannan antigen) in conjunction with compatible clinical and radiographic findings. Probable IFD requires the presence of a host factor, a clinical criterion, and a mycological criterion. Possible IFD is defined as sufficient clinical evidence of IFD without any mycological support. Cases that met the criterion for a host factor and a clinical criterion but for which mycological criteria were absent were considered as possible IFD.

If proven, probable, or possible IFD was diagnosed during the administration of micafungin prophylaxis, the case was recorded as a treatment failure. Another antifungal agent could be administered along with micafungin, or patients could be switched to other antifungal agents. Treatment success was defined as the absence of proven, probable, or possible IFD up to 4 weeks after therapy.

### 2.5. Statistical Analysis

The treatment success rate was calculated using two-sided exact, with 95% confidence intervals by using Fisher’s exact test. The expected treatment success rate with micafungin (73%) was higher than that of oral itraconazole (60%) [16,20]. This percentage was set as a standard value for the current study. With 5% of one-sided type I error and 80% of power, 102 patients were required. Considering an exclusion rate of 10%, a total of 112 patients were required.

Patient characteristics and safety were analyzed for the ITT full-analysis set. The primary endpoint was analyzed in the primary endpoint efficacy set.

## 3. Results

### 3.1. Enrollment

**Figure 2:**
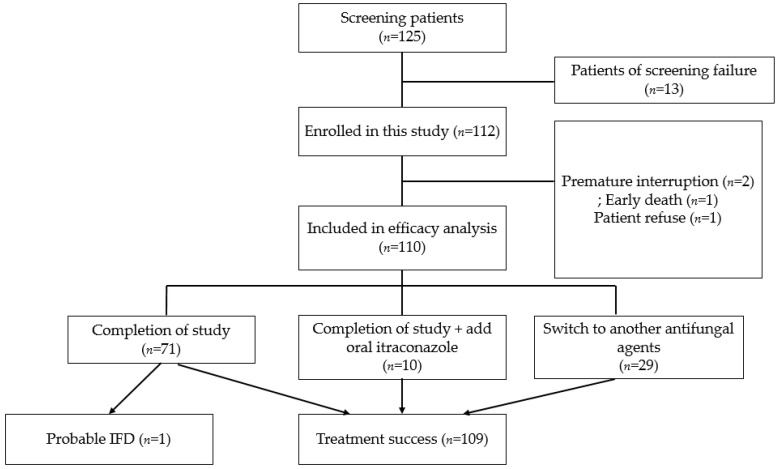
Flow diagram of the study.

From November 2011 to February 2017, 125 patients were screened from Seoul National University Hospital in Korea. A total of 13 patients were excluded owing to screening failure, and 112 patients were enrolled (Figure 2). Patient characteristics are listed in Table 1.

### 3.2. Treatment Outcome

The study protocol was completed without premature interruption in 110 patients (98.2%). The reasons for interruption in micafungin treatment included early death (*n* = 1) and patient refusal (*n* = 1). The median duration of micafungin prophylaxis was 16 days (range, 2–26 days). Of 110 patients in the efficacy group, 10 were treated with oral itraconazole because of a positive galactomannan result without evidence of IFD, and micafungin was replaced by another antifungal agent in 29 with persistent fever despite administration of broad-spectrum antibiotics. Treatment success was achieved in 109 patients (99.1%, 95% exact confidence lower limit: 97.4%). A total of 38 out of 110 patients had positive results of galactomannan Ag test. Since there were no patients with clinical or radiographic findings, it was not diagnosed as IFD only with a positive galactomannan result. Probable IFD was diagnosed in one patient who had consolidative infiltration and nodules on chest CT in addition to a positive galactomannan result 27 days after completion of the study protocol. Lung biopsy was performed; however, there was no evidence of IFD. This patient was administered intravenous itraconazole and other broad-spectrum antibiotics and recovered approximately 2 weeks after the administration of other antifungal agents. Twenty-nine patients with persistent fever in which micafungin was replaced by another antifungal agent had fever for median 11 days (range 6–16 days), and there was no incidence of IFD. No patients were diagnosed with possible or proven IFD.

### 3.3. Adverse Events

In the full-analysis set, 21 patients (18.8%) experienced 22 AEs during the study protocol; however, all AEs were classified as “unlikely” related to micafungin. Nine patients (8.0%) experienced grade III AEs, and no patient experienced grade IV AEs. None of the patients discontinued micafungin administration owing to AEs (Table 2). No deaths were reported related to the study drug. All patients succeeded in engraftment. Neutrophil and platelet engraftment was performed on day 11 (range, 9–19) and day 18 (range, 10–38), respectively.

### 3.4. Mortality

There was no case of IFD-related mortality. However, one patient in the premature interruption group had persistent fever during the conditioning regimen and died of septic shock 5 days after HSCT. There were no deaths related to the study drug.

## 4. Discussion

Previous studies have reported an IFD incidence of 12–22.5% among allogeneic [21] and 8–10% among autologous [22] HSCT recipients. The risk of IFD is lower in autologous HSCT than in allogeneic HSCT; however, the incidence of IFD in autologous HSCT has been reported. In a recent study, Linke et al. reported a cumulative IFD incidence of 8.7% in 95 pediatric patients who underwent autologous HSCT [7]. IFD is a major cause of morbidity and mortality in pediatric patients who have previously undergone HSCT [23,24]. The reasons for increasing risk of IFD include prolonged neutropenia, immunosuppressive therapy, delayed immune reconstitution, use of indwelling catheters, and broad-spectrum antibiotics [4,23]. Pagano et al. [24] reported that there could be fatal cases after autologous HSCT in adult patients. For high-risk patients, such as HSCT recipients, antifungal agents were initiated at a period of high risk of infection to prevent fungal infections. There are several evidence-based guidelines for adults undergoing HSCT [25]. However, there are no evidence-based guidelines for pediatric patients, and only few reports have described its prophylactic use in pediatric patients.

In recently suggested guidelines for antifungal prophylaxis in pediatric patients undergoing allogeneic or autologous HSCT, Michelle et al. [26] recommended that patients (1 month to <19 years of age) who have previously undergone allogeneic or autologous HSCT with anticipated neutropenia for >7 days should be administered intravenous or oral fluconazole (6–12 mg/kg/day; maximum 400 mg/day) from the start of conditioning.

In the trial by Van Burik et al. [15], which included allogeneic or autologous HSCT in 39 pediatric and 386 adult patients, a higher proportion of patients receiving micafungin had successful prophylaxis. The overall efficacy of micafungin was superior to that of fluconazole as an antifungal prophylaxis during the neutropenic phase after HSCT (80.0% in the micafungin arm versus 73.5% in the fluconazole group; *p* = 0.03). However, the sample size of the pediatric group in this study was small, and the enrolled population was heterogeneous, including autologous and allogeneic HSCT recipients.

In patients with fungal infections after allogeneic or autologous HSCT, causative species included *Candida* (51%) and *Aspergillus* (25%) [22]; however, fluconazole treatment did not show efficacy against *Aspergillus* species. Among other antifungal agents, itraconazole has greater activity and coverage, including aspergillosis, blastomycosis, coccidioidomycosis, histoplasmosis, and paracoccidioidomycosis, than fluconazole [27]. Itraconazole undergoes extensive metabolism via the cytochrome P450 3A4 enzyme system and increases the interactions with many other medications, including drugs important to HSCT recipients, such as cyclosporine, tacrolimus, and antineoplastic agents [28]. Huang et al. compared the efficacy of micafungin with itraconazole in patients aged 18–70 years with neutropenia who underwent allogeneic or autologous HSCT. The study reported that the treatment success rate of micafungin was similar to that of itraconazole, with significant differences in the incidence of drug-related AEs between the micafungin and itraconazole groups (8% versus 26.5%; *p* = 0.00, chi-square test).

Amphotericin B is a polyene antifungal agent with in vitro activity against a wide variety of fungal pathogens [29]. However, adverse effects are common with the usage of amphotericin B, especially nephrotoxicity, being the most serious, occurring early in the course of treatment [30]. There are a few studies on amphotericin B as a prophylactic antifungal agent during allogeneic or autologous HSCT in pediatric patients. Roman et al. [31] used liposomal amphotericin B, which is a lipid formulation of the antifungal agent, to lessen the toxicity of conventional amphotericin B. It could efficiently prevent IFD; however, it induced grade 3–4 nephrotoxicity in 7/57 (12%) patients, and in 6/57 (11%), liposomal amphotericin B was discontinued due to toxicity.

Micafungin is a member of the echinocandins and a semisynthetic lipopeptide synthesized by a chemical modification of a fermentation product of Coleophoma, which has a broad spectrum of fungicidal activity and is associated with minimal toxicity [12]. Drug interactions are expected to be uncommon with micafungin. Because micafungin is metabolized in the liver and not metabolized by the CYP 450 system [32]. It has mostly been used for the treatment of invasive candidiasis in pediatric populations and suggested in the 2009 guideline as an alternative therapy to fluconazole in the prophylaxis of patients with a standard risk against fungal infections, namely those with allogeneic HSCT or those with prolonged neutropenia and mucosal damage after autologous HSCT [25]. There are several studies including phase I studies that provide information regarding the dose regimens of micafungin for pediatric patients [33,34]. An initial dose of 1–2 mg/kg/day is usually recommended, and 1 mg/kg/day (for patients < 50 kg body weight) was effective in a phase III study that compared micafungin with fluconazole in pediatric HSCT recipients. Micafungin achieved a treatment success rate of 69.2%, whereas fluconazole at 8 mg/kg/day achieved a treatment success rate of 53.3% [15]. Park et al. [17] demonstrated the effectiveness of micafungin as an IFD prophylactic during neutropenia in children and adolescents who underwent allogeneic HSCT. Of 132 patients, treatment success with micafungin was achieved in 119 (90.2%). There were no proven fungal infections in any patient, and none of them experienced grade IV AEs.

The known AEs of micafungin in children include diarrhea, epistaxis, abdominal pain, headache, nausea, vomiting, fever, chills, elevation of alanine aminotransferase/aspartate aminotransferase values, hypokalemia, thrombocytopenia, mucositis, and rash [35]. Our research also showed similar trends as described in previous studies. AEs included nausea, vomiting, diarrhea, and elevation of alanine aminotransferase/aspartate aminotransferase values, and all AEs were self-limiting and adjustable. However, there were no AEs related to the administration of micafungin, and none of the patients discontinued micafungin owing to AEs.

There has been no prospective study on the safety and utility of antifungal prophylaxis in pediatric patients with autologous HSCT. This is the first prospective study on the prophylaxis of IFD using micafungin in autologous HSCT. This study demonstrated that micafungin is a safe and effective option for antifungal prophylaxis in pediatric patients receiving autologous HSCT, with promising efficacy without significant AEs in a larger cohort than those described in other studies.

## Figures and Tables

**Table 1:** Characteristics of the patients.

Number of patients	112
Median age, Years (range)	8 (1–19)
Sex, No. (%)	
Male	67
Female	45
Diagnosis, No. (%)	
Non-Hodgkin lymphoma	16
Hodgkin lymphoma	4
Medulloblastoma	16
Atypical teratoid rhabdoid tumor	7
Neuroblastoma	15
Primitive neuroectodermal tumor	7
Osteosarcoma	14
Ewing sarcoma	6
Germ cell tumor	6
Others (acute leukemia, retinoblastoma, Wilms tumor, pineoblastoma, rhabdoid tumor of kidney, choroid plexus carcinoma)	21
Neutrophil engraftment, days (range)	11 (7–19)
Platelet engraftment, days (range)	18 (10–38)

**Table 2:** Non-hematologic adverse events during study protocol in 112 patients.

Adverse Events	Grade I	Grade II	Grade III
Gastrointestinal			
Abdominal pain	0	0	0
Nausea	2	2	6
Vomiting	2	4	0
Diarrhea	1	0	1
Constipation	0	0	0
Hepatic			
ALP increased	0	0	0
ALT increased	0	3	1
AST increased	0	3	1
Bilirubin increased	0	0	0
Electrolyte imbalance			
Hypocalcemia	1	2	0
Hypokalemia	0	0	1
Hypomagnesemia	0	0	0
Hyponatremia	0	1	0
Total	6	15	10

## Data Availability

The data presented in this study are available from the corresponding author on a reasonable request.
